# Retrospective Case-Control Study Genes Related to Bone Metabolism That Justify the Condition of Periodontal Disease and Failure of Dental Implants in Patients with down Syndrome

**DOI:** 10.3390/ijms24097723

**Published:** 2023-04-23

**Authors:** María Baus-Domínguez, Raquel Gómez-Díaz, Daniel Torres-Lagares, Jose-Luis Gutiérrez-Pérez, Guillermo Machuca-Portillo, María-Ángeles Serrera-Figallo

**Affiliations:** 1 Department of Dentistry, Faculty of Dentistry, University of Seville, 41009 Seville, Spain; danieltl@us.es (D.T.-L.); gmachuca@us.es (G.M.-P.); 2 Institute of Biomedicine of Seville, 41013 Seville, Spain; rgomez-ibis@us.es; 3 Oral and Maxillofacial Unit, Virgen del Rocio Hospital, 41013 Seville, Spain; jlgp@us.es; 4 Oral Surgery Department, Faculty of Dentistry, University of Seville, 41009 Seville, Spain

**Keywords:** implantology, peri-implantitis, periodontal disease, down syndrome, osseointegration, inflammation, genes

## Abstract

Down syndrome patients show success rates in dental implants much lower than those observed in the general population. This retrospective case-control study aimed to identify possible genes that are related to the regulation of inflammatory responses and bone metabolism related to periimplantitis and implant loss, as well as genes related to bone quality. This process involved using the functional analysis of the gene expression software Transcriptome Analysis Console (TAC version 4.0 Applied Biosystems^TM^, Thermo Fisher Scientific, Waltham, MA, USA) and a search for possible candidate genes involved. The focus was placed on the 93 genes related to periodontitis, periimplantitis, bone loss, implant loss, and genes related to bone quality and regulators underlying the establishment and maintenance of osseointegration. Five genes showed statistically significant results (*p* < 0.05) in our comparison. Four of them, IL1B (*p* = 0.023), IL1RN (*p* = 0.048), BGLAP (*p* = 0.0372) and PTK2 (*p* = 0.0075) were down-regulated in the periodontal disease and implant rejection group, and only one was overexpressed: FOXO1A (*p* = 0.0552). The genes with statistically significant alterations described in this article determine that the group of Down syndrome patients with periodontal disease and implant failure is a group of patients genetically susceptible to suffering from both conditions together.

## 1. Introduction

According to the United Nations (UN), Down syndrome, a genetic alteration caused by the existence of a chromosome with more than pair 21, has an estimated worldwide incidence of 1 in 1100 newborns.

Oral problems in these patients continue to be a great challenge for dentists. It is well known that patients with Down syndrome have a higher prevalence of periodontal disease compared to the general population [1,2,3,4,5], which, together with the higher prevalence of agenesis (30–81% excluding third molars) [2,3], can become completely edentulous from a very young age, where the only therapeutic alternative to address quality of life is the placement of dental implants. 

However, patients with Down syndrome have a dental implant success rate ranging from 50–100% reported in series and case reports with a short follow-up period [1,3], where the most extensive case series exhibited survival of 76.7% [1], showing that the same article has a survival of 84.5%. Retrospective case-control studies, such as the one by Corcuera-Flores et al. 2016 [4] with a 4-year follow-up, showed that of the 31 implants placed in patients with Down syndrome, 29% were lost and all presented marginal bone loss. This survival rate is much lower than that of the general population, for whom a systematic review and meta-analysis estimates a survival of 96.4% at 10 years (95% CI: 95.2–97.5%) [6].

In line with the above, another systematic review and meta-analysis in the population with medical pathologies reports an overall survival of 97.3% at one year and 96.1% at five years [7], where the greatest involvement was found in patients who had received radiation therapy or were under antiresorptive treatment. It is true that this study does not report data on implant survival in patients with mental disabilities, but refers to studies on survival rates in patients with Parkinson’s disease ranging between 82.1% and 100% [7,8]. Together with the results of the study by Corcuera et al. to 2016 [4], where they also monitored dental implants in patients with cerebral palsy, among whom 36% presented with marginal bone loss but no failed implant placements, it can continue to be observed that dental implant success rates in patients with motor disorders or mental disabilities are higher than those observed in patients with Down syndrome.

Most published studies refer to the fact that implant failure in patients with Down syndrome is due to alteration of the immune response in combination with their cognitive disability and other oral characteristics of these patients, such as previous history of periodontal disease, macroglossia, parafunctional habits or osteoporotic-type alveolar bone, among others [1,2,3,8,9,10]. However, it has been reported that most failures occur during the osseointegration period, before implant loading [1,2,3,8]. In addition to the systematic review by Contaldo et al., 2021 [5], which reports the results of the comparison between patients with Down syndrome, patients with mental or motor disabilities, and patients without mental or motor disorders, patients with Down syndrome significantly present with the worst periodontal clinical indices, leading us to believe that there must be a little-studied cause underlying implant failure in this type of patient. 

Since 2019, our research group has been documenting the complex gene network hidden behind this process. The results identified genes with a statistically significantly altered expression, such as the Metallothionein genes and their relationship with bone metabolism [11,12], genes related to alterations in the inflammatory response of Down syndrome patients with periodontal disease vs. without periodontal disease [13], and genes related to alterations in the inflammatory response and the subsequent influence on implant failure in Down syndrome patients with periodontal disease and implant failure vs. Down syndrome patients with periodontal disease and positive evolution of dental implants [14].

In the present study, we examine whether patients with Down syndrome exhibit statistically significant differences in expression of genes related to bone metabolism, when comparing groups of patients with Down syndrome who present with periodontal disease to patients with Down syndrome who have not had a history of periodontal disease. In this study, we are investigating genes involved in the regulation of inflammatory responses and bone metabolism that are related to peri-implantitis and implant loss, as well as genes related to bone quality, the transient chondrogenic phase, the vitamin D axis, and the peripheral circadian rhythm of regulators underlying the establishment and maintenance of osseointegration.

## 2. Results

### 2.1. Patients and Characteristics

Due to strict inclusion and exclusion criteria, only 11 patients were included in the study. As such, after publishing our first results of the gene expression analysis on metalothioneins [11], we validated the results that were published last June 2022 [12], where we demonstrated that despite the small sample, conclusive results can be obtained.

All patients were Spanish. Four of them were diagnosed with periodontal disease and failure of dental implants, while seven patients did not have periodontal disease and had a positive evolution of their dental implants at two years of evolution (Table 1).

### 2.2. Gene Expression Analyses

Of the 93 genes studied in our gene expression analysis through the software Transcriptome Analysis Console (TAC version 4.0 Applied Biosystems^TM^, Thermo Fisher Scientific, Waltham, MA, USA), only five genes showed statistically significant (*p* < 0.05) altered results when comparing Down syndrome patients with periodontal disease and dental implant failure (PD+IR+) to Down syndrome patients without periodontal disease and with a positive evolution of their dental implants (PD−IR−). 

These genes were IL1B, IL1RN, OCN (BGLAP), FOXO1, and PTK2, with four of them showing a down-regulated result and two of them showing an up-regulated result (Table 2).

### 2.3. Functional Analyses of Differentially Expressed Genes

Each of the five genes that showed statistically significant differential expression when comparing both groups were studied in the same manner as described in the article Differential expression of inflammation-related genes in Down syndrome patients with or without Periodontal Disease [12]. For the analysis, the databases of the National Center for Biotechnology Information (NCBI) and Online Mendelian Inheritance in Man^®^ were used. Information on the metabolic pathways in which genes participate was obtained from the Kyoto Encyclopaedia of Genes and Genomes (KEGG) and Reactome for those genes whose information was not available in the KEGG (Table 3).

According to these databases, only IL1B and IL1RN were observed to share the cytokine-cytokine receptor interaction pathway. The rest of the genes do not appear to share cellular pathways.

## 3. Discussion

In an article previously published by our group [13], it was observed when comparing patients with Down syndrome with periodontal disease (DS+PD+) to patients with Down syndrome without periodontal disease (DS+PD−) that despite suffering from the same syndromic condition, both groups did not show the same susceptibility to periodontal disease, instead showing differential expression of *TNFSF13B*, *ITGB2*, *ANXA5* and *ANXA3*, of the 92 inflammation-related genes presented in the TaqMan^TM^ Array Plate Human Inflammation Kit (Thermo Fisher Scientific, Waltham, MA, USA). Similarly, in a subsequent study [14] in which the study groups were modified to Down syndrome patients with periodontal disease and implant failure (PD+IR+) compared to Down syndrome patients with periodontal disease and without implant failure (PD+IR−), both groups showed clear signs of active periodontal disease, and yet the results were totally different. Of the 92 inflammation-related genes present in the 96-plex card (TaqMan^TM^ Array Plate Human Inflammation Kit (Thermo Fisher Scientific, Waltham, MA, USA)), six genes provided statistically significant results (*p* < 0.05) and none matched those of the previous [13] (*PLCG2*, *ALOX5*, *LTAH4*, *VCAM1*, *PLA2G2A* and *PLA2G10*).

Taking this into account, and considering that when searching for and selecting Down syndrome patients who met the inclusion and exclusion criteria defined above, no Down syndrome patient was observed who did not have periodontal disease but did have implant failure (PD−IR+). This is an important result to take into account. We ask ourselves, how is it possible for two groups of individuals with Down syndrome who, by definition, present with disorders in the immune system and in the inflammatory response to develop different degrees of periodontal and/or periimplant involvement that lead to loss of dental implant (PD+IR+) while others do not present with any signs of active periodontal disease and show a positive evolution of their dental implants (PD−IR−)?

To try to answer this question, we focused on those genes that, according to the consulted bibliography, seem to have a greater relationship with osseointegration, bone metabolism, bone healing, osteoblasts, osteoclasts, etc. collected in Table 4 and Table 5, together with those obtained from the manual search previously mentioned.

Proinflammatory cytokines such as interleukins are essential biochemical mediators for the control of a correct inflammatory response. Among them, IL-1 appears to be a useful biomarker in the diagnosis of periimplantitis, since it plays an important role in the pathogenesis of periodontitis [16,17], being one of the ten most important molecules [16], and in the pathogenesis of immune-inflammatory processes [17]. Interleukin 1-Beta is involved in various cellular and tissue functions, including osteoclast differentiation. The *IL1B* gene, which codes for IL-1B, shows a statistically significant (*p* = 0.023) down-regulated expression in our results. It may be that since IL-1B is a pro-inflammatory cytokine, its downregulation is positive for the clinical situation of periodontal disease. However, this is known to not completely be the case, since pro-inflammatory cytokines, including IL-1, are crucial for the regulation of RANKL expression in osteoblasts related to inflammation that can affect bone metabolism [18].

Similarly, a recent study showed that the P2X Prurinergic Receptor encoded by the *P2RX5* gene is essential for the proper production of IL-1B by osteoclasts and is necessary for their maturation [18]. In our study group, it is observed how PD+IR+ patients show an up-regulation of *P2RX5* (FD = 1.29; *p* = 0.0377), which could explain the clinical condition of active periodontal disease and implant failure in this group of patients, despite showing down-regulation of *IL1B*, since the study by Kim et al. 2018 showed in an in vivo model of how *P2RX5*-deficient mice were protected against LPS-induced inflammatory bone loss.

Similarly, the IL-1 receptor antagonist gene, *IL1RN*, encodes IL-Ra that inhibits IL-1B activity by competitive binding to IL-1 receptors [19,20,21,22], which acts as an anti-inflammatory cytokine and may even favour osteogenic differentiation of gingival-derived mesenchymal stem cells (GMSC) [21], also shows statistically significant down-regulated expression (*p* = 0.048) in the group of PD+IR+ patients. This result is especially interesting, since IL-1Ra has been described to protect GMSC cell viability and osteogenic capacity through the irruption of the NF-κB signaling pathway mediated by TLR-4 and activated by P. gingivalis-LPS [21]. Therefore, IL-1Ra is considered a strong defence against periodontitis, whose deficiency influences the most serious destruction of periodontal tissue in a murine experimental model of periodontitis [20]. Previous studies showed that mice without IL-1Ra have greater colonization of Aggregatibacter Actinomycetemcomitans, one of the bacteria most associated with incisor-molar [22] pattern periodontitis, formerly known as aggressive periodontitis, and from which patients with Down syndrome often suffer. Both are periodontal pathogenic bacteria, one of the most abundant species in the microbiome of Down syndrome patients compared to other patients with mental disabilities and periodontitis but who do not have Down syndrome, as reported by the systematic review and meta-analysis by Contaldo et al. 2021 [5]. Therefore, it is emphasised that poor motor skills are not the only cause responsible for the higher prevalence of periodontitis and its earlier and more severe form [5].

Despite the fact that our study group shows the down-regulated *IL1B* gene, since, according to studies, inadequate or even increased IL-1Ra secretion seems not to be enough to counteract the deleterious effects of IL-1B [20], we could speculate that the other way around, the lower expression of IL-1B is not important enough to not observe the deleterious effects of this proinflammatory cytokine in patients who also show reduced expression of IL-1Ra, dependent on a regulation to loss of its *IL1RN* encoded gene.

However, *BGLAP*, better known as osteocalcin (*OCN*), which encodes the most abundant non-collagenous calcium binding protein in mineralised tissues [23,24,25], also shows statistically significant down-regulation in our study group (*p* = 0.0372). This protein plays an important role in bone resorption and mineralisation [23,24,25]. It might be thought that having a lower expression of the gene encoding the aforementioned protein implies an alteration in the osseointegration period of implants, since under physiological conditions during the bone healing period of the implants, changes in the OCN levels are observed, and this protein being used as a biomarker of osseointegration is documented in several studies [24]. Consistent with this fact, decreased levels of OCN were also observed in smokers with periodontitis [26].

*PTK2*, Protein-Tyrosine Kinase, Cytoplasmic presents, like the previous ones, with a statistically significantly decreased expression (*p* = 0.0075). This gene encodes the focal adhesion protein tyrosine kinase (FAK), an important molecule in cell adhesion signaling. The deletion of FAK does not appear to affect bone development dependent on mature osteoblasts, but it does appear to hinder bone healing, since its activation is extremely important for osteogenic differentiation [24]. In vivo [27], it was observed that the absence of FAK is related to regeneration, defective bone, slower extracellular matrix deposition, and larger bony callus formation. Furthermore, the results of the study warn that the extracellular matrix of osteoblasts in *PTK2* mutants is not conducive to osteoclast attachment [27], which implies the alteration of correct bone remodelling.

The fact that patients with PD+IR+ Down syndrome show decreased expression of the *PTK2* gene, together with the inflammatory environment that characterises this group of patients, may cause FAK levels to be greatly reduced, since FAK is inactivated in the presence of inflammatory conditions, which implies lower differentiation of osteoblasts by direct action of a compromise in their adhesion [25].

Lastly, *FOXO1A*, Forkhead Box O1A, encodes a protein that is a member of the class O forkhead box family, proteins involved in bone cell function, which seems to be related to certain bone diseases such as osteoporosis or osteoatritis [28]. *FOXO1A* controls osteogenesis by regulating oxidative stress (homeoastis redox), among other processes such as glucose metabolism, ageing, or adipogenesis of mesenchymal stem cells and osteoblast precursors, optimising RUNX2 function [28]. In a study of mice with overexpression of *FOXO1A* [28], researchers observed a 17.2% higher bone-implant contact. However, in our study, *FOXO1A* is observed to be up-regulated in the study group with a statistically significant difference (*p* = 0.0552) and a clinical condition of periodontal disease and implant failure, a result contrary to that shown in this study, but consistent with what the same research group previously documented in diabetic mice [29,30], where osseointegration was enhanced by inhibiting *FOXO1A*, probably by enhancing abnormal glucose metabolism, resulting in FOXO1 probably having different biological effects under normal conditions than in patients with oxidative stress induced by hyperglycemia. This is consistent with the systemic hyperglycemia condition that many Down syndrome patients present with [31], which leads us to believe that the PD+IR+ Down syndrome patients in our study have a worse potential for osteogenic differentiation and therefore a lower volume decreased bone formation and trabeculae formation, related to its alteration of *FOXO1A* gene expression together with its alteration in glucose metabolism.

When proposing a study to explain and understand the genetic influence on the development of certain pathologies—in our case, periodontitis and periimplant diseases in patients with Down syndrome—it is necessary to identify which patients are involved in such research, since it is well known that ethnicity determines differences in genotype. The literature affirms that there is no gene that can be used throughout the world’s different ethnic groups to determine susceptibility to developing periodontal diseases [16]. It is very likely that more than a dozen genes are involved in these diseases [16]. The more genes that are involved, the greater the complexity of the process behind this event.

As we have stated in other articles, the objective we pursue is not to explain the failure of implants in Down syndrome patients, but rather to shed light on the complex process that leads to their high failure rate in dental implantology, to elucidate which ones are candidates for the placement of dental implants, and thus be able to offer them quality treatment.

On the other hand, we are still aware of the limitation of working with a sample made up of 11 patients. However, this does not prevent us from obtaining valid and reproducible results, as we have already demonstrated in our gene validation study [12].

Similarly, we consider it interesting to propose a future study in which both groups are diagnosed with periodontal disease, but only one of them presents failure in dental implant treatment.

## 4. Materials and Methods

### 4.1. Type of Study

This is a retrospective case-control study approved by the Ethics Committee of the Hospital Virgen del Rocío (File PI-0081-2016) that complies with all the guidelines of the Declaration of Helsinki of the World Medical Association, Ethical Principles for Medical Research Involving Human Subjects [32].

This is a descriptive and observational study in which the only invasive procedure was a dental examination and the extraction of a peripheral blood sample to obtain genetic material from the patients.

All patients, or the person responsible for them, received an information sheet and gave their informed consent based on the direct benefits of the research for the patient.

### 4.2. Samples and Groups

The study groups consisted of patients with Down syndrome diagnosed with periodontal disease (PD+) at the time of the clinical examination for this study together with implant failure/rejection (IR+) at two years of evolution (PD+IR+) and Down syndrome patients who did not suffer from periodontal disease (PD−) and presented a positive evolution of their dental implants (IR−) after two years of evolution (PD−IR−) (PD+IR+ vs. PD−IR−).

The exclusion criteria were as follows:

- Patients who did not have Down syndrome
- Patients who received treatment that could potentially affect bone metabolism
- Patients treated with short implants
- Patients rehabilitated with immediate loading
- Patients with active or untreated periodontal disease at the time of implant placement
- Patients with implants who had a progression period of less than two years.
- Patients for whom there were no data at the time of dental implant placement or at 2 years of progression.

The diagnosis of periodontal disease, as well as the evolution of implants, was made based on the clinical history of the patients and the comparison of panoramic radiographs (immediate postoperative vs. two years of evolution) to calculate the marginal bone loss (MBL) and differentiate whether implant-related bone defects were due to misplacement or bone loss resulting from periimplantitis.

The patients in the control group (PD−IR−) had not been diagnosed with periodontal disease at any time in their life and had no bleeding on probing (BoP) at the time of clinical examination. However, the patients in the study group (PD+IR+) who, if they were diagnosed with periodontal disease at some of their visits, it was treated and inactive at the time of dental implant placement or the patient had previously lost all teeth.

Bassani et al. [33] developed the criteria used to determine whether a patient had periodontal disease or not. According to this criterion, periodontal disease is defined by the presence of 3 or more teeth with 1 or more sites with a loss of clinical attachment level (CAL) greater than or equal to 3 mm, in combination with the existence of BoP in the mentioned sites.

However, in our study, we do not reflect the numerical data that refer to pocket depth measurements and/or loss of attachment to the remaining teeth of the patients since these data are not of interest to our study, since our variable “periodontal disease (PD)” was taken as a dichotomous variable (yes (PD+)/no (PD−)), as was implant failure (yes (IR+)/no (IR−)).

On the other hand, to calculate the MBL, the Lagervall and Jansson index [34] was used and validated for use in this type of study by Corcuera-Flores et al. [35]. This method divides implants into four groups based on their MBL:

- Grade 0: implants with no marginal bone loss.
- Grade 1: marginal bone loss of one third or less of the total length of the implant.
- Grade 2: one-third, but less than two-thirds, of the total length of the implant.
- Grade 3: marginal bone loss greater than two thirds of the total length of the implant.
- A fifth group (Grade 4) of those patients who lost the implant was added.
- In our study, a failed implant (RI+) is understood as lost at two years of follow-up, or periimplant bone loss of at least grade 2 on the Lagervall and Jansson scale.

We retrieved demographic and clinical data from patients’ medical records and verified all essential data.

### 4.3. Sampling and total RNA Isolation

After dental clinical examination, the patients included in the study underwent two blood samples that were collected in PAXgene^TM^ tubes (reference 762,165) to obtain genetic material for RNA. The samples were transported to the Institute of Biomedicine of Seville (IBiS), refrigerated at 2–8 °C for up to 5 days. Tubes were stored at −20 °C or −80 °C as appropriate.

RNA samples were extracted using PAXgene^TM^ BLOOD miRNA KIT (reference 763,134) from QIAGEN. The extraction was carried out in the QIAcube automated station of the same brand.

First, the amount of RNA was quantified using a visible light spectrophotometer with Thermo Nanodrop 2000C equipment to ensure that the samples were well processed prior to storage. We continued with the quantification of the samples selected for this study using a much more precise measurement, fluorescence, through Thermo Qubit 3.0 equipment (reference Q33,216; Singapore, Malaysia) from Invitrogen^TM^ by Life Technologies (Thermo Fisher Scientific, Waltham, MA, USA).

All the data referring to the quantification of the genetic material were included in a database.

### 4.4. Functional Analysis of Expressed Genes

The selected RNA was amplified and hybridised using the Gene-Chip^®^ WT PLUS reagent kit (Thermo Fisher Scientific, Santa Clara, CA, USA). Amplification was carried out from an initial total of 55 nanogrammes (ng) of RNA, and followed the indications described in the GeneChip^®^ WT PLUS Reagent Kit manuals.

The platform chosen for this study was the Thermo Scientific GeneChip^®^ Scanner 3000, and the chosen chips were Clariom S solutions for humans, mice, and rats, with more than 20,000 genes entered to measure expression levels.

Scanning with the Thermo Scientific GeneChip^®^ Scanner 3000 was performed following the protocols for loading matrix cartridges. Finally, the analysis was performed by normalising and using the robust multiple array (RMA) method, and the analysis of the different gene expressions was performed using the software (Transcriptome Analysis Console (TAC version 4.0 Applied Biosystems^TM^, Thermo Fisher Scientific, Waltham, MA, USA).

### 4.5. Statical Analysis from the Reference Articles

The analysis of the different gene expressions was carried out using software (Transcriptome Analysis Console (TAC version 4.0 Applied Biosystems^TM^, Thermo Fisher Scientific, Waltham, MA, USA). The reference genes taken for the search in our study were the genes related to the modulation of the inflammatory response and bone metabolism from the review by Xun Chen and Yu Zhao [36] (Table 4).

The search was extended by adding the genes from the Critical Reviews of I. Nishimura [15] (Table 5).

More genes were added as a result of the search of updated bibliography in PubMed using the keywords “implant loss”, “bone loss”, “dental implant”, “osseointegration”, “genes or genetics”, “peri-implantitis”, “periodontitis”, and “bone metabolism”. So, manually, the following genes were added to the search: LTA, LTB, IL8, IL22, CCL3, OSTERIX, P4H, P3H1, P3H2, P3H3, PLOD1, PLOD2, PLOD3, CRTAP, PPIE, HAPLN1, PTGS2, MMP8, RUNX2, OPG, IRF8, EXH2, KDM1A, HDAC2, HDAC7, HDAC9, IBSP, ALPL, FOXO1, VDR, PI3K, AKT, LEF1, PPARG2, PTK2, ITGA1 and ITGB3, making a total of 93 genes studied.

## 5. Conclusions

Despite the fact that all patients have the same syndromic condition, it is observed that the patients of the study group (PD+IR+) show an altered expression of genes that participate, via very different pathways, in bone metabolism (IL1B, IL1RN, PTK2, BGLAP, FOXO1A). This is able to explain the loss of dental implants from the point of view of alteration in bone healing and osseointegration. However, this response should not only explain the failure condition or not of dental implants, since it also works with the periodontal disease variable, which is not an independent variable of dental implant failure, but is joint in this study. For this reason, we conclude that the genes described in this article determine the PD+IR+ group as a genetically susceptible group to suffer from both conditions together.

## Figures and Tables

**Table 1:** Clinical data of the patients included in the study. To increase the number of patients in the control group, we included Patients 9, 10, and 11, who did not yet have implants in place. However, these patients did not have periodontal disease and would therefore not be candidates for early failure after implant placement.

Patient	Group	Age	Sex	Smoker	Drinker	History of Controlled Periodontal Disease	Implants Placed	Bone Regeneration	Result after Two Years of Follow-Up
1	PD+IR+	41	F	No	No	Yes	2	No	1 implant lost and 1 implant with severe peri-implantitis
2	PD+IR+	39	F	No	No	Yes	3	No	1 implant lost and 2 implants with severe peri-implantitis
3	PD+IR+	33	M	No	No	Yes	4	No	2 implants with severe peri-implantitis
4	PD+IR+	35	M	No	No	Yes	12	No	3 implants lost
5	PD−IR−	40	F	No	No	No	3	No	No implant failure or peri-implantitis
6	PD−IR−	34	F	No	No	No	2	No	No implant failure or peri-implantitis
7	PD−IR−	43	F	No	No	No	3	No	No implant failure or peri-implantitis
8	PD−IR−	48	F	No	No	No	2	No	No implant failure or peri-implantitis
9	PD−IR−	44	M	No	No	No	No	No	No implant failure or peri-implantitis
10	PD−IR−	38	M	No	No	No	No	No	No implant failure or peri-implantitis
11	PD−IR−	44	M	No	No	No	No	No	No implant failure or peri-implantitis

Demographic data were not significant and do not influence the results shown.

**Table 2:** Results of the analysis of differential gene expression in the two study groups: Down syndrome patients with periodontal disease and implant failure (PD+RI+) and Down syndrome patients without periodontal disease and with positive evolution of the implants. (PD−RI−), both after two years of evolution. The asterisk before the number is the ID format presented by OMIM.

Gene	Gene ID-OMIM	Gene Name	Related to	PD+IR+ AVG (log2)	PD−IR− AVG (log2)	PD+IR+ Standard Deviation	PD−IR− Standard Deviation	Fold Change	*p* Value	Cytogenetic Location
IL1B	* 147720	Interleukin 1-Beta	Periodontal Disease Peri-Implantitis Bone Loss Implant Loss	12.9	13.72	0.37	0.66	−1.76	0.023	2q14.1
IL1RN	* 147679	Interleukin 1 Receptor Antagonist	Periodontal Disease Peri-Implantitis Implant Loss	9.31	10.06	1.04	1.6	−1.68	0.048	2q14.1
BGLAP	* 112260	Gamma-Carboxyglutamic Acid Protein, Bone	Osteoblasts	8.09	8.45	0.66	0.38	−1.28	0.0372	1q22
FOXO1A	* 136533	Forkhead Box O1a	Osteoblasts	7.7	7.22	0.17	0.46	1.4	0.0552	13q14.11
PTK2	* 600758	Protein-Tyrosine Kinase, Cytoplasmic	Osteoblasts	5.44	6.26	0.5	0.43	−1.77	0.0075	8q24.3

**Table 3:** Summary of the cellular pathways in humans of each of the genes with a statistically significantly altered result.

Gene	Cellular Pathways in Humans (kegg)	*p* Value	Fold Change
IL1B	(1) Antifolate resistance (2) MAPK signaling pathway (3) Cytokine-cytokine receptor interaction (4) NF-kappa B signaling pathway (5) Necroptosis (6) Osteoclast differentiation (7) Toll-like receptor signaling pathway (8) NOD-like receptor signaling pathway (9) Cytosolic DNA-sensing pathway (10) C-type lectin receptor signaling pathway (11) Hematopoietic cell lineage (12) IL-17 signaling pathway (13) Th17 cell differentiation (14) TNF signaling pathway (15) Inflammatory mediator regulation of TRP channels	0.023	−1.76
IL1RN	Cytokine-cytokine receptor interaction	0.048	−1.68
BGLAP	Parathyroid hormone synthesis, secretion, and action (regulation of bone mass)	0.03722	−1.28
FOXO1A	(1) FoxO signaling pathway (2) AMPK signaling pathway (3) Longevity regulating pathway (4) Cellular senescence (5) Insulin signaling pathway (6) Thyroid hormone signaling pathway (7) Glucagon signaling pathway (8) Insulin resistance—Homo sapiens (human)	0.0552	1.4
PTK2	(1) Endocrine resistance (2) ErbB signaling pathway (3) Chemokine signaling pathway (4) PI3K-Akt signaling pathway (5) Axon guidance (6) VEGF signaling pathway (7) Focal adhesion (8) Leukocyte transendothelial migration (9) Regulation of actin cytoskeleton (10) Growth hormone synthesis, secretion, and action	0.0075	−1.77

**Table 4:** Genes related to the regulation of the inflammatory response and bone metabolism that lead to peri-implantitis and implant loss.

Periodontitis	Peri-Implantitis	Bone Loss	Implant Loss
*TGFB1*, *MMP1*, *MMP9*, *BRINP3*, *IL6*, *IL1A*, *IL1B*, *IL1RN*, *IL2*, *IL10*, *TNFA*, *IL4*, *TLR2*, *TLR4*	*IL17*, *BMP4*, *BRINP3*, *IL1A*, *CD14*, *IL1RN*, *IL1B*, *TNFA*, *RANK*, *RANKL*, *FGF3*	*IL1A*, *IL1B*, *CALCR*	*IL1A*, *IL1B*, *ILRN*, *IL2*, *IL4*, *IL6*, *IL10*, *MMP1*, *MMP9*, *TNFA*, *TGFB1*, *RANKL*

**Table 5:** Genes related to bone quality, transient chondrogenic phase, vitamin D axis, and peripheral circadian rhythm of regulators underlying the establishment and maintenance of osseointegration, from the review by I. Nishimura [15].

Cartilage-Related Genes	Cartilage or Bone-Related Genes	Bone-Related Genes
*ASPN*, *ACAN*, *Col2a1*, *CHAD*, *CILP*, *Col9a2*, *Col9a3*, *Col10a1*, *Col11a1*, *CRTAP*, *DSPG3*, *HAPLN1*, *HAPLN3*, *PANX3*, *SOX9*, *TNS1.*	*BGN*, *BMP6*, *BMPR1A*, *DCN*, *DMP1*, *FMOD*, *FN*, *ICAM1*, *SMAD5/SMAD9*, *SMAD6/SMAD7.*	*Col5A1*, *MGP*, *OCN*, *OMD*, *ON*, *OPN*

## Data Availability

The data presented in this study are available on request from the corresponding authors.
